# Sleep apnea is not associated with worse outcomes in kidney transplant recipients

**DOI:** 10.1038/srep06987

**Published:** 2014-11-11

**Authors:** Katalin Fornadi, Katalin Zsuzsanna Ronai, Csilla Zita Turanyi, Tushar S. Malavade, Colin Michael Shapiro, Marta Novak, Istvan Mucsi, Miklos Z. Molnar

**Affiliations:** 1 Dept. of Neurology, Semmelweis University, Budapest, Hungary; 2 Institute of Behavioral Sciences, Semmelweis University, Budapest, Hungary; 3 Department of Medicine, Division of Nephrology, University Health Network, University of Toronto, Toronto, Canada; 4 Dept. of Psychiatry, University Health Network, University of Toronto, Toronto, Canada; 5 Dept. of Ophthalmology, University Health Network, University of Toronto, Toronto, Canada; 6 Institute of Pathophysiology, Semmelweis University, Budapest, Hungary; 7 Division of Nephrology, Department of Medicine, University of Tennessee Health Science Center, Memphis, TN, United States

## Abstract

Obstructive sleep apnea(OSA) is one of the most common sleep disorders in kidney transplant recipients, however its long-term consequences have only rarely been investigated. Here, we hypothesized that the presence of OSA would be associated with higher risk of mortality and faster decline of graft function in kidney transplant recipients. In a prospective cohort study 100 prevalent kidney transplant recipients who underwent one-night polysomnography at baseline and were followed for a median 75 months. Generalized linear mixed-effects models and Cox regression models were used to assess the association between OSA and the rate of progression of chronic kidney disease(CKD) and mortality. The estimated slopes of estimated glomerular filtration rate(eGFR) in patients with and without OSA were compared using a two-stage model of eGFR change including only OSA as a variable. In this model patients with OSA (eGFR versus time was −0.93 ml/min/1.73 m^2^/yr(95%CI:−1.75 to−0.11) had a similar slope as compared to patients without OSA(eGFR versus time was −1.24 ml/min/1.73 m^2^/yr(95%CI: −1.67 to −0.81). In unadjusted Cox proportional regression analyses OSA was not associated with higher all-cause mortality risk (Hazard Ratio(HR) = 1.20; 95% Confidence Interval(CI): 0.50–2.85). No association was found between the presence of OSA and the rate of progression of CKD or all-cause mortality in prevalent kidney transplant recipients.

Obstructive sleep apnea (OSA) is one of the most clinically important forms of sleep-related breathing disorders. The prevalence of moderate and severe obstructive OSA (AHI ≥ 15 and the presence of daytime symptoms of OSA) is 2–4% in the general population^1^. OSA is associated with increased cardiovascular morbidity and mortality^2^,^3^. OSA is reportedly associated with higher risk of stroke, hypertension, diabetes mellitus, congestive hearth failure, arrhythmias and the metabolic syndrome and also with fatal and non-fatal cardiovascular (CV) events^4^,^5^,^6^,^7^.

Previous studies have shown high prevalence of OSA (16–54%) in patients with chronic kidney disease (CKD)^8^,^9^, indicating that OSA is more common in hemodialyzed patients than in general population^10^. Earlier we reported a similarly high prevalence of OSA in kidney transplant recipients compared to waitlisted dialysis patients^11^. However, Mallamaci et al. found that OSA was not more common in kidney transplant recipients than age, body mass index (BMI) and gender matched individuals from general population^12^.

Despite the potential clinical relevance, the long-term consequences of OSA have only rarely been investigated among kidney transplant recipients. The association between OSA and hypertension, accelerated atherosclerosis and vascular damage was confirmed in patients with CKD^13^. The complex pathophysiology that links OSA to CV risk may also have a detrimental effect on renal function. Indeed, Kinebuchy et al. documented glomerular hyperfiltration in patients with OSA, which was alleviated by short-term continuous positive airway pressure (CPAP) treatment suggesting that OSA may be a risk factor of progressive renal dysfunction^14^.

We designed this prospective cohort study to determine the association between presence of OSA and long-term outcome, such as the decline of graft function and all-cause mortality in a randomly selected sample of stable, prevalent kidney transplant recipients. OSA was assessed using polysomnography at baseline. Based on the previous findings we hypothesized that the presence of OSA would be associated with faster decline of graft function and higher risk of mortality.

## Results

### Demographic data and baseline characteristics of the sample

The SLeep disorders Evaluation in Patients after kidney Transplantation (SLEPT) - study cohort was described previously^11^. The basic characteristics (age, gender, estimated glomerular filtration rate (eGFR), blood hemoglobin, serum albumin) of the 100 participating transplant (Tx) patients (“Tx study sample”) were similar to the characteristics of the “total clinic population” (Figure S1). Baseline patient characteristics are shown in Table 1. Eighty-five percent of the patients were taking steroids, 43% were administered cyclosporine A microemulsion formulation (CsA), 71% were on mycophenolate-mofetil (MMF), 46% patients were administered tacrolimus and 5% were on azathioprine. Only 1% and 12% of the patients took everolimus and sirolimus, respectively. Six percent of our patients had at least one previous transplantation.

The percentage of male patients was significantly higher among patients with versus without apnea-hypopnea index (AHI) ≥ 15/h. The prevalence of mild (5/h ≤ apnea-hypopnea index (AHI) < 15/h), moderate (15/h ≤ AHI < 30/h) and severe OSA (AHI ≥ 30/h) was 18%, 11% and 14% in our kidney transplant recipients^11^. We did not find any association between OSA and the level of education, tobacco use, comorbidity or age (Table 1). However, patients with versus without OSA had significantly higher body mass index (BMI), neck- and abdominal circumference. Patients with versus without OSA had similar eGFR. While serum albumin and C-reactive protein (CRP) levels were similar in the two groups, the blood hemoglobin level was higher in patients with OSA versus without OSA (Table 1). The median transplant vintage, the median dialysis vintage and cumulative end stage renal disease (ESRD) time were all similar in patients with versus without OSA (Table 1). Donor characteristics (gender, type and age) and transplant related variables (cold ischemic time, cumulative acute rejection rate, panel reactive antibody (PRA), delayed graft function (DGF) and human leucocyte antigen (HLA) mismatches) were similar in patients with versus without OSA (not shown). None of the immunosuppressive medications was significantly associated with the presence of OSA (not shown).

### Decline of graft function

**Figure 1:**
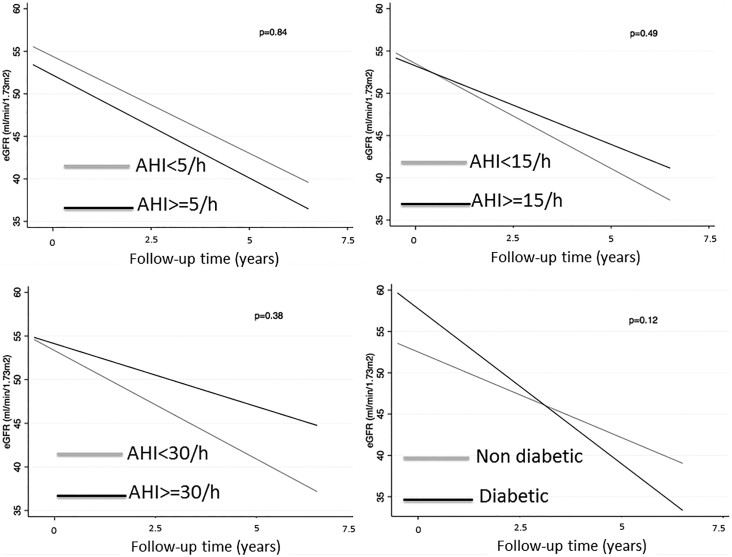
Rate of graft function loss in patients with and without OSA using 5/h (panel A), 15/h (panel B) and 30/h (panel C) as cut-off and in diabetic patients (panel D).

In a multilevel mixed-effects model of change of eGFR, the overall slope of eGFR versus time was −1.17 ml/min/1.73 m^2^/yr (95% CI: −1.55 to −0.78) (Table 2). Figure 1 depicts the estimated slopes of eGFR in patients with and without OSA using different AHI cut-off level based on a two-stage model of eGFR change. OSA was only included as a stage 2 variable, showing that patients with OSA (eGFR versus time was −0.93 ml/min/1.73 m^2^/yr (95% CI: −1.75 to −0.11) had a similar slope than patients without OSA (eGFR versus time was −1.24 ml/min/1.73 m^2^/yr (95% CI: −1.67 to −0.81) (Table 2). Similar results were found using different cut-off levels for OSA (Table 2). However, patients with diabetes showed more rapid decline of graft function than non-diabetic counterparts (Table 2 and Figure 1 Panel D). Additionally, eGFR slope was similar in patients with high versus low desaturation index (Table S2). Similar results were found in males and females (Table S3).

In addition, neither the presence of OSA (as a categorical variable, defined by various cut-off values) nor the AHI index (as a continuous variable) was associated with the rapid decline of eGFR using >4 ml/min/1.73 m^2^/year as a cut-off level (Table 3). Similar result was found in a sensitivity analysis using >6 ml/min/1.73 m^2^/year (Table S1), >2 ml/min/1.73 m^2^/year (not shown) or >8 ml/min/1.73 m^2^/year as a cut-off level for rapid decline (not shown). A similar result was found when we adjusted for baseline eGFR (not shown). Moreover, neither the high desaturation index (as a categorical variable, defined by cut-off 5/hour) nor the desaturation index (as a continuous variable) was associated with the rapid decline of eGFR using >4 ml/min/1.73 m^2^/year (Table S2). Similar results were found in males and females (Table S3).

### All-Cause Mortality

**Figure 2:**
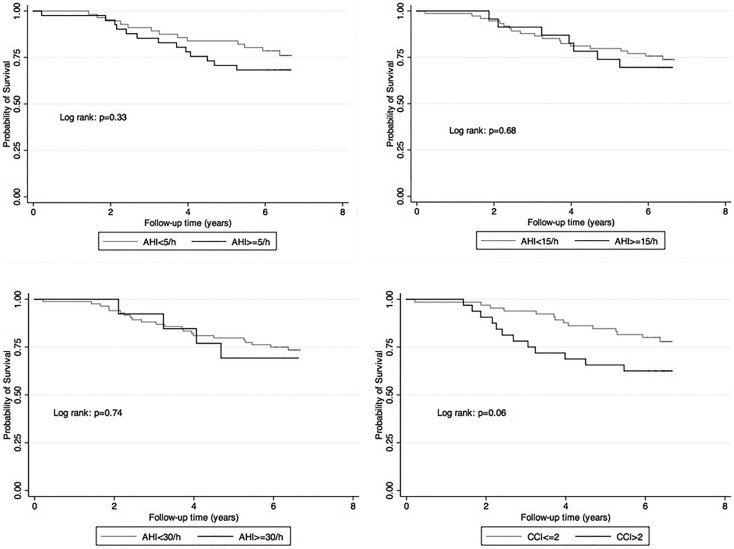
Presence of OSA using 5/h (panel A), 15/h (panel B) and 30/h (panel C) as cut-off and comorbidity (panel D) and all-cause mortality.

Of the 100 participants 26 patients died and none were lost to follow-up during a median follow-up of 75 months. The crude all-cause mortality rate (including deaths with functioning graft and deaths after returning to dialysis) was 47.8/1000 patient-years (95% confidence interval [CI]: 32.5–70.1). The unadjusted mortality rate was similar among patients with and without OSA (crude mortality rates in the AHI ≥ 15/h group: 54.7/1000 patient-years (95%CI: 26.1–114.7); AHI < 15/h group: 45.6/1000 patient-years (95%CI: 29.1–71.5); p = 0.68). Time to death was also similar in patients with versus without OSA, as shown on the Kaplan-Meier plot (Figure 2 Panel B). We found similar results using different cut-off levels for OSA (Figure 2 Panel A and Panel C). Patients with higher burden of comorbidity (Charlson Comorbidity Index > 2), however, had higher unadjusted mortality rate than patients with less comorbidities (Figure 2 Panel D).

Table 4 shows the association of all-cause mortality with OSA in 100 kidney transplant recipients. In unadjusted Cox proportional regression analyses AHI ≥ 15/h was not associated with higher all-cause mortality risk (Hazard Ratio (HR) = 1.20; 95% Confidence Interval (CI): 0.50–2.85). Similar results were found using different cut-off levels for OSA or using AHI as continuous predictor variable (Table 4). However, in our unadjusted Cox proportional regression analyses a 1-point higher Charlson Comorbidity Index significantly predicted all-cause mortality (HR_1 point increase_ = 1.28; 95% CI: 1.04–1.58) (Table 4).

Moreover, neither the high desaturation index (as a categorical variable, defined by cut-off 5/hour) nor the desaturation index (as a continuous variable) was associated with the all-cause mortality (Table S2). Similar results were found in males and females, however there was an increased trend for higher risk of all-cause death in females (Table S3).

### Combined Outcome

Table 5 shows the association of combined outcome (death, return to dialysis or rapid decline) with OSA in our cohort. In unadjusted logistic regression analyses AHI ≥ 15/h was not associated with higher combined outcome risk (Odds Ratio (OR) = 0.95; 95% CI: 0.38–2.35). Similar results were found using different cut-off levels for OSA or using AHI as continuous predictor variable (Table 5). However, in our unadjusted logistic regression analyses presence of diabetes was significantly associated with higher risk of combined outcome (OR = 4.24; 95% CI: 1.30–13.89) (Table 5). Moreover, neither the high desaturation index (as a categorical variable, defined by cut-off 5/hour) nor the desaturation index (as a continuous variable) was associated with combined outcome (Table S2).

## Discussion

In this prospective cohort study, which is one of the largest studies using polysomnography in kidney transplant recipients, there was no association between the presence of OSA and the rate of decline of graft function in prevalent kidney transplant recipients. In addition, we could not find any association between presence of OSA and all-cause mortality in this population.

In this dataset the prevalence of OSA was higher than published by Mallamaci et al. recently^12^. One potential reason for these discordant results may be the different methodology used. Mallamaci et al. report the utilization of polygraphy and cardiorespiratory recording while we used standard polysomnography with EEG. Another explanation may be the differences in the study population. Our sample was older, had more diabetics, higher BMI and had somewhat worse mean eGFR.

The rate of decline of graft function was similar in transplant recipients with and without OSA. This is surprising as previous data showed strong correlation between severity of CKD and prevalence of OSA in CKD population^15^, although the directionality of the association could not be established. However, several reports suggested that this might be a consequence of fluid overload or nocturnal rostral fluid overshift in these patients^16^,^17^,^18^. Further studies are needed to assess whether the presence of OSA contributes to renal dysfunction or progressive loss of kidney function or it is only fluid overload associated with declining renal function that leads to the increasing prevalence of OSA in association with worse kidney function. Another potential explanation is the fact that the renal graft is denervated. Previous studies suggested that renal denervation, a potential treatment of therapy resistant hypertension, had positive effect on OSA^19^ and could potentially be utilized as an alternative treatment for OSA^20^. One can speculate, that the potential negative effect of OSA on kidney function is mediated via the relative sympathetic overactivation which is transmitted to the kidney via its sympathetic nervous supply. This could not be operational in the transplanted kidney.

We previously showed that high risk of OSA is an independent predictor of graft loss among female kidney transplant recipients^21^. However, we used questionnaire to assess the risk of OSA in our patients. No study was performed to assess the reliability of this questionnaire in kidney transplant recipients, however previous study showed that questionnaire can be unreliable in patients with kidney disease to assess sleep disorders^22^. Further studies are needed to assess the reliability of these questionnaires in kidney transplant recipients.

There was no association between OSA and all-cause mortality or combined outcome in our patients. OSA is an independent predictor of mortality in the general population^2^,^3^, and overnight hypoxemia^23^ and OSA^24^ are independent predictors of cardiovascular events in dialysis patients. Moreover, we previously showed that the presence of OSA was associated with increased cardiovascular risk in this population^11^. However, in our analysis we used the Framingham risk score to assess cardiovascular risk^11^, which is not accurate to predict cardiovascular risk in transplant population. One potential explanation could be the lack of excessive daytime sleepiness (EDS) in our patients with OSA^25^. As in the case of our transplant recipients, there was very weak correlation between EDS and severity of OSA in patients on maintenance hemodialysis^26^. A previous study showed that there was no association between all-cause mortality and OSA in elderly patients without EDS^27^. In addition, some studies have suggested that EDS may be an important element in understanding the clinical significance of OSA^28^,^29^,^30^, although the exact mechanism that could link EDS and clinical outcomes is unknown. Finally, it is possible that our study lacked the sufficient power to detect a modest difference of the outcomes of interest between patients with versus without OSA.

Limitations of this report should be noted. The prospective cohort design precludes any causal conclusions. Determining sample size was driven mainly by feasibility, and no formal sample size calculations had been done before the study. *Post hoc* power calculations suggest that this study was powered to detect *0.25 or lower hazard ratio* of the mortality between the non OSA versus OSA groups with a *power of 80%*, but the power is insufficient to detect a smaller difference. Based on this data, we can conclude that our study was underpowered and further, larger studies are needed to confirm or reject our results. Patients from a single center were enrolled; therefore, our results are not to be generalized without further considerations. Finally, a substantial proportion of transplant recipients refused to participate (Figure S1). Importantly, we did not find any difference between participants *versus* nonparticipants; therefore, it is unlikely that refusal introduced a systematic bias that would distort our conclusions significantly. Refusal rate was similar in other studies that used polysomnography in end stage renal disease population^10^,^31^. We also acknowledge that there is a potentially “unavoidable” selection bias that affects all studies of sleep disorders that are based on polysomnography, such as ours: only motivated or symptomatic patients accept the stress of polysomnography, whereas “good sleepers” may opt to avoid this test. We cannot exclude the presence of this bias in our study.

## Conclusion

This is the first report to present data from a large number of transplant recipients regarding the association between OSA and long-term clinical outcomes, such as decline of graft function and all-cause mortality in kidney transplant recipients. The rate of progression of graft function and all-cause mortality were similar in transplant recipients with and without OSA. Further, larger studies are needed to confirm or reject our results before we can make any recommendation for screening and treatment of OSA in kidney transplant recipients.

## Methods

### Sample of patients and data collection

For this study (“SLeep disorders Evaluation in Patients after kidney Transplantation (SLEPT) Study”) potentially eligible patients were selected from all prevalent adult transplant recipients (“total clinic population; n = 1214) who were regularly followed at a single outpatient transplant center on December 31, 2006. After applying exclusion criteria (previous diagnosis of OSA, recent start (less than 3 months) on dialysis or transplant, active and acute respiratory disorder, acute infection, hospitalization within 1 month, surgery within 3 months) 1198 patients remained (“base population”; n = 1198). From this “base population” we randomly selected and approached 150 patients (“Tx study sample”) using the simple random sampling strategy offered by SPSS 15.0 (Figure S1). From these 150 patients 100 patients agreed to participate. They underwent one overnight polysomnography and were followed for a median of 6 years. Detailed history including age, gender, level of education, tobacco use and etiology and history of CKD were collected at enrolment.

### Polysomnography

Standard, attended overnight polysomnography was performed in our sleep laboratory (SOMNOscreen™ PSG Tele, SOMNOmedics GmBH, Germany, CE0494). Recordings were manually scored by two somnologists. Sleep stages were determined in 30 s epochs according to Rechtschaffen and Kales^32^. Apnea was defined as the absence of airflow for more than 10 s; hypopnea was defined as a clearly discernible reduction in airflow for more than 10 s associated with an arousal and/or reduction in oxygen saturation >3%^33^. The AHI was defined as the number of apneas and hypopneas per hour of sleep. Similarly to previous publications^34^,^35^ the term ‘OSA’ refers to moderate or severe apnea (AHI ≥ 15) in this paper, unless stated otherwise. Desaturation index was defined as the number of desaturations per hour of sleep. We used 5/hour for definition of high versus low number of desaturation index.

### Laboratory data

Laboratory, demographic, anthropometric, medication data and single pool (sp) Kt/V were extracted from the medical records. Estimated glomerular filtration rate (eGFR) was calculated using the abbreviated Modification of Diet in Renal Disease (MDRD) study formula^36^.

### Comorbidity

Comorbidity was assessed by the modified Charlson Comorbidity Index (CCI)^37^,^38^ completed by the transplant physician responsible for the given patient. We also collected data about coronary disease and hypertension from the medical records.

### Transplantation and donor related data; immunosuppressive therapy

Transplantation related information collected included current medications, transplant and dialysis “vintage” (i.e. time elapsed since transplantation or since the initiation of dialysis treatment), time spent on dialysis prior to transplantation, type of transplantation (deceased donor or living donor), history of cumulative acute rejection, HLA mismatch, titer of pre-transplant panel reactive antibodies (PRA), cold ischemic time (CIT), age and gender of donor and history of delayed graft function. Time elapsed since the initiation of the first treatment for ESRD (cumulative ESRD time) was also calculated. Standard maintenance immunosuppressive therapy generally consisted of prednisolone, either CsA or tacrolimus, combined with MMF or azathioprine, everolimus or sirolimus.

### Follow-up

Patients were followed for an average 75 months (median, [interquartile range - IQR]: 75.2 [69.9–77.0] months). The primary outcome variable was deterioration of graft function. We collected each patient's eGFR in every 6 months for the entire follow-up period. If a patient was started on dialysis or re-transplanted or eGFR was less than 15 ml/min/1.73 m^2^ the last value was the last available eGFR or the last value below 15 ml/min/1.73 m^2^ and no further data was collected. The “rapid decline of graft function” status was defined if the deterioration of eGFR was higher than 4 ml/min/1.73 m^2^/year. The secondary outcome variable was all-cause mortality, which included all deaths with a functioning graft and deaths occurring after the graft failure (i.e. after initiation of dialysis). Deaths and re-initiations of maintenance dialysis were ascertained from the hospital database. Deaths were validated by cross-referencing with data from the Hungarian Central Office of Administrative and Electronic Public Service, which is the government agency maintaining official vital status records. In addition, we also performed a sensitivity analysis using combined outcome, defined as death of any cause or return to dialysis or rapid decline of graft function (deterioration of eGFR was higher than 4 ml/min/1.73 m^2^/year) of graft function. None of our patients were treated for OSA at baseline or during the follow-up period, however CPAP treatment was offered all of them.

### Ethical approval

The study was approved by the Ethics Committee of the Semmelweis University (4/2007). Before enrolment, patients received detailed verbal and written information about the aims and protocol of the study and signed an informed consent. All experiments were performed in accordance with relevant guidelines and regulations.

### Statistical Analysis

Statistical analyses were carried out using the STATA 12.1 software. Results are presented as percentage, mean (±standard deviation, SD) or medians (interquartile range, IQR). Continuous variables were compared using Student's t-test or the Mann-Whitney U test and categorical variables were analyzed with chi-square test. In all statistics, two-sided test were used and the results were considered statistically significant if p was less than 0.05.

For multivariate analysis, logistic- and Cox regressions were applied. Independent variables were included in the multivariate models based on theoretical considerations. Variance influence factors (VIF) were used to indicate collinearity between independent variables. The association between baseline AHI level and all-cause mortality was assessed using Cox proportional regression analysis and Kaplan-Meier plots with log rank test. Proportional hazards assumptions were tested using scaled Schoenfeld residuals.

The association between the presence of OSA (using different AHI cut-off levels) and the slopes of eGFR versus time were examined in generalized linear mixed-effects models allowing for a random intercept and slope using the “XTMIXED” command in STATA. The change in eGFR from baseline until death, start of dialysis, or re-transplantation (whichever occurred first) was studied in all transplant recipients who had at least three serum creatinine measurements (n = 100; median number of measurments = 8, range: 3 to 14) by using a two-stage model formulation. In such a model, the level 1 change describes intra-individual changes in eGFR, and the level 2 model describes how the change coefficients differ across participants. The covariate of interest (AHI as independent variable) is thus included in the level 2 models to explain interindividual differences in intra-individual change (slope).

## Supplementary Material

Supplementary Information Supplemental tables and figure

## Figures and Tables

**Table 1:** Patients' characteristics at baseline

	Patients with AHI ≥ 15/h (n = 25)	Patients with AHI < 15/h (n = 75)	p value
**Demographic parameters:**			
Male (%)	80	49	0.01
Age (mean ± SD) (years)	54 ± 12	50 ± 13	0.15
Level of education (%):	24	20	0.10
Primary education or less	24	12	
Skilled workers	12	40	
High school or equivalent	40	28	
University diploma			
**Anthropometric parameters:**			
Neck circumference (mean ± SD) (cm)	40 ± 3	37 ± 4	<0.01
Abdominal circumference (mean ± SD) (cm)	107 ± 12	95 ± 15	<0.01
BMI (mean ± SD) (kg/m^2^)	29 ± 5	26 ± 5	<0.01
**Comorbidities:**			
Tobacco use (%)	20	20	1.00
Prevalence of diabetes (%)	16	20	0.66
Prevalence of hypertension (%)	100	89	0.09
Charlson Comorbidity Index (median; IQR) (point)	2; 0	2; 1	0.22
Prevalence of coronary heart disease (%)	8	8	1.00
Prevalence of congestive heart failure (%)	8	8	1.00
Prevalence of peripheral vascular disease (%)	12	12	1.00
Prevalence of cerebro-vascular disease (%)	4	1	0.41
Prevalence of atrial fibrillation (%)	8	1	0.09
**Blood Pressure:**			
Average of systolic blood pressure (mean ± SD) (mmHg)	147 ± 21	139 ± 18	0.06
Average of diastolic blood pressure (mean ± SD) (mmHg)	85 ± 13	83 ± 11	0.50
**Laboratory parameters:**			
Blood Hemoglobin (mean ± SD) (g/L)	141 ± 17	132 ± 16	0.02
Serum albumin (mean ± SD) (g/L)	40 ± 4	40 ± 3	0.72
Serum CRP (median; IQR) (mg/l)	3.8; 4.3	2.8; 4.6	0.43
eGFR at baseline (mean ± SD) (ml/min./1.73 m^2^)	51 ± 18	52 ± 19	0.63
**History of ESRD:**			
Transplant “vintage” (median; IQR) (months)	60; 109	67; 78	1.00
Dialysis “vintage” (median; IQR) (months)	25; 39	18; 28	0.24
Cumulative ESRD time (median; IQR) (months)	117; 147	96; 85	0.18
**Medications:**			
ACE inhibitors (%)	8	24	0.08
Any type of anti-hypertensive drug (%)	92	96	0.63
Hypnotic drugs (%)	8	20	0.17
**Sleep parameters:**			
Epworth Sleepiness scale (median; IQR) (point)	4 (5)	5 (5)	0.24
Average oxygen saturation during sleep (mean ± SD) (%)	91.8 ± 1.6	94.0 ± 2.0	<0.01
Slow wave sleep (median; IQR) (%)	9 (11)	12 (11)	0.41

**Table 2:** Rate of graft function loss in patients with and without OSA – univariate analysis

	eGFR changes (ml/min/1.73 m^2^/year)	Confidence interval of eGFR changes (ml/min/1.73 m^2^/year)	p-value
**All patients**	−1.17	(−1.55)–(−0.78)	N/A
**Patients with AHI < 5/h**	−1.14	(−1.65)–(−0.63)	0.84
**Patients with AHI ≥ 5/h**	−1.21	(−1.80)–(−0.62)	
**Patients with AHI < 15/h**	−1.24	(−1.67)–(−0.81)	0.49
**Patients with AHI ≥ 15/h**	−0.93	(−1.75)–(−0.11)	
**Patients with AHI < 30/h**	−1.24	(−1.67)–(−0.81)	0.38
**Patients with AHI ≥ 30/h**	−0.72	(−1.44)–(−0.01)	
**Absence of diabetes**	−1.04	(−1.46)–(−0.61)	0.12
**Presence of diabetes**	−1.88	(−2.56)–(−1.19)	

**Table 3:** Predictors of rapid progression (>4 ml/min/1.73 m^2^/year) of graft function – multivariable analysis

*Model 1*	*Odds ratio (OR)*	*Confidence interval of OR*	*p-value*
**AHI ≥ 5/h (vs AHI < 5/h as reference)**	1.45	0.60–3.49	0.41
**Presence of diabetes (vs absence of diabetes as reference)**	2.10	0.72–6.13	0.17
**Serum albumin (+1 g/dl increase)**	0.89	0.78–1.02	0.09
**Age (+10 year increase)**	0.98	0.68–1.41	0.91

*Model 2*	*Odds ratio (OR)*	*Confidence interval of OR*	*p-value*
**AHI ≥ 15/h (vs AHI < 15/h as reference)**	0.93	0.35–2.51	0.89
**Presence of diabetes (vs absence of diabetes as reference)**	2.08	0.72–6.02	0.18
**Serum albumin (+1 g/dl increase)**	0.89	0.78–1.01	0.08
**Age (+10 year increase)**	1.01	0.71–1.44	0.94

*Model 3*	*Odds ratio (OR)*	*Confidence interval of OR*	*p-value*
**AHI ≥ 30/h (vs AHI < 30/h as reference)**	0.40	0.10–1.63	0.20
**Presence of diabetes (vs absence of diabetes as reference)**	2.12	0.72–6.22	0.17
**Serum albumin (+1 g/dl increase)**	0.89	0.78–1.02	0.10
**Age (+10 year increase)**	1.05	0.73–1.50	0.80

*Model 4*	*Odds ratio (OR)*	*Confidence interval of OR*	*p-value*
**AHI (+1/h increase)**	0.99	0.96–1.02	0.58
**Presence of diabetes (vs absence of diabetes as reference)**	2.06	0.71–5.96	0.18
**Serum albumin (+1 g/dl increase)**	0.89	0.78–1.02	0.08
**Age (+10 year increase)**	1.03	0.72–1.48	0.86

**Table 4:** All-cause mortality and presence of sleep apnea – univariate analysis

	Hazard ratio (HR)	Confidence interval of HR	p-value
**AHI ≥ 5/h (vs AHI < 5/h as reference)**	1.47	0.68–3.16	0.33
**AHI ≥ 15/h (vs AHI < 15/h as reference)**	1.20	0.50–2.85	0.68
**AHI ≥ 30/h (vs AHI < 30/h as reference)**	1.20	0.41–3.48	0.74
**AHI (+1/h increase)**	1.01	0.98–1.03	0.62
**Charlson Comorbidity Index (+1 point increase)**	1.28	1.04–1.58	0.02

**Table 5:** Combined outcome (death or graft loss or rapid progression (>4 ml/min/1.73 m^2^/year) of graft function) and presence of sleep apnea – univariate analysis

	Odds ratio (OR)	Confidence interval of OR	p-value
**AHI ≥ 5/h (vs AHI < 5/h as reference)**	1.44	0.65–3.19	0.37
**AHI ≥ 15/h (vs AHI < 15/h as reference)**	0.95	0.38–2.35	0.91
**AHI ≥ 30/h (vs AHI < 30/h as reference)**	0.62	0.20–1.95	0.42
**AHI (+1/h increase)**	1.00	0.97–1.03	0.68
**Presence of diabetes (vs absence of diabetes)**	4.24	1.30–13.89	0.02
