# Supplementary material for: Sleep apnea is not associated with worse outcomes in kidney transplant recipients
Source: Sci Rep. 2014 Nov 11;4:6987. doi: 10.1038/srep06987 (PMC5381499; doi:10.1038/srep06987)

SREP-14-05435.R1

Title:

Sleep apnea is not associated with worse outcomes in kidney transplant recipients

Authors:

Katalin Fornadi MD, PhD[1*], Katalin Zsuzsanna Ronai MD[2*], Csilla Zita Turanyi MD[2], Tushar S Malavade, MD, MBBS, DNB[3], Colin Michael Shapiro BSc, FRCPC, MBBCH, PhD[4,5], Marta Novak MD, PhD[2,4], Istvan Mucsi MD, PhD[2,3,6], Miklos Z Molnar MD, PhD[7]

From:

[1]Dept. of Neurology, Semmelweis University, Budapest, Hungary; [2]Institute of Behavioral Sciences, Semmelweis University, Budapest, Hungary; [3]Department of Medicine, Division of Nephrology, University Health Network, University of Toronto, Toronto; [4]Dept. of Psychiatry, University Health Network, University of Toronto, Toronto, Canada; [5]Dept. of Ophthalmology, University Health Network, University of Toronto, Toronto, Canada; [6]Institute of Pathophysiology, Semmelweis University, Budapest, Hungary; [7]Division of Nephrology, Department of Medicine, University of Tennessee Health Science Center, Memphis, TN, United States

*** these authors contributed equally**

SUPPLEMENT TABLES

Table S1: Predictors of rapid progression (>6 ml/min/1.73 m^2/year) of graft function – multivariate analysis

Model 1	***Odds ratio (OR)***	***Confidence interval of OR***	***p-value***
**AHI≥5/h (vs AHI<5/h as reference)***	0.67	0.25-1.85	0.44
Model 2	***Odds ratio (OR)***	***Confidence interval of OR***	***p-value***
**AHI≥15/h (vs AHI<15/h as reference)***	0.97	0.32-2.92	0.96
Model 3	***Odds ratio (OR)***	***Confidence interval of OR***	***p-value***
**AHI≥30/h (vs AHI<30/h as reference)***	0.47	0.09-2.35	0.36
Model 4	***Odds ratio (OR)***	***Confidence interval of OR***	***p-value***
**AHI (+1/h increase)***	0.99	0.96-1.02	0.50

*Adjusted for age, presence of diabetes and serum albumin level

Table S2: Association between desaturation index and outcomes

	eGFR changes (ml/min/1.73 m^2/year)	*Confidence interval of eGFR changes (ml/min/1.73 m^2/year)*	*p-value*
**Patients with Desaturation index <5/h**	-1.04	(-1.59) - (-0.48)	0.46
**Patients with Desaturation index ≥5/h**	-1.30	(-1.83) - (-0.76)	
*Model**	*Odds ratio (OR) of rapid progression (>4 ml/min/1.73 m^2/year) of graft function*	*Confidence interval of OR*	*p-value*
**Desaturation index ≥5/h (vs Desaturation index <5/h as reference)**	1.54	0.64-3.73	0.34
**Desaturation index (+1/hour)**	0.99	0.97-1.02	0.59

*Model***	*Hazard ratio (HR) of all-cause mortality*	*Confidence interval of HR*	*p-value*
**Desaturation index ≥5/h (vs Desaturation index <5/h as reference)**	1.61	0.73-3.56	0.24
**Desaturation index (+1/hour)**	1.01	0.98-1.03	0.57
*Model**	*Odds ratio (OR) of combined outcome*	*Confidence interval of OR*	*p-value*
**Desaturation index ≥5/h (vs Desaturation index <5/h as reference)**	1.02	0.42-2.46	0.97
**Desaturation index (+1/hour)**	0.99	0.97-1.02	0.56

*: Model adjusted for age, serum albumin and presence of diabetes

**: Unadjusted model

Table S3: Association between OSA and outcomes in females and males

	eGFR changes (ml/min/1.73 m^2/year)	*Confidence interval of eGFR changes (ml/min/1.73 m^2/year)*	*p-values*
	Females		p for gender interaction: 0.72
**AHI ≥15/h**	-0.23	(-2.87) - (2.40)	0.51
**AHI <15/h**	-1.24	(-1.84) - (-0.63)	
	Males		
**AHI ≥15/h**	-0.95	(-1.79) - (-0.11)	0.46
**AHI <15/h**	-1.33	(-1.94) - (-0.72)	
Model*	***Odds ratio (OR) of rapid progression (>4 ml/min/1.73 m^2/year) of graft function***	***Confidence interval of OR***	***p-values***
	Female		p for gender interaction: 0.43
**AHI ≥15/h (vs AHI <15/h as reference)**	0.59	0.49-6.99	0.67
	Male		
**AHI ≥15/h (vs AHI**	0.79	0.23-2.70	0.71

**<15/h as reference)**			
Model**	***Hazard ratio (HR) of all-cause mortality***	***Confidence interval of HR***	***p-values***
	Female		p for gender interaction: 0.36
**AHI ≥15/h (vs AHI <15/h as reference)**	4.36	0.87-21.82	0.07
	Male		
**AHI ≥15/h (vs AHI <15/h as reference)**	0.63	0.23-1.78	0.38
Model*	***Odds ratio (OR) of combined outcome***	***Confidence interval of OR***	***p-value***
	Female		p for gender interaction: 0.80
**AHI ≥15/h (vs AHI <15/h as reference)**	4.44	0.38-51.96	0.24
	Male		
**AHI ≥15/h (vs AHI <15/h as reference)**	0.52	0.15-1.77	0.29

*: Model adjusted for age, serum albumin and presence of diabetes

**: Unadjusted model

SUPPLEMENT FIGURE

Figure S1: Flow chart of the patient selection

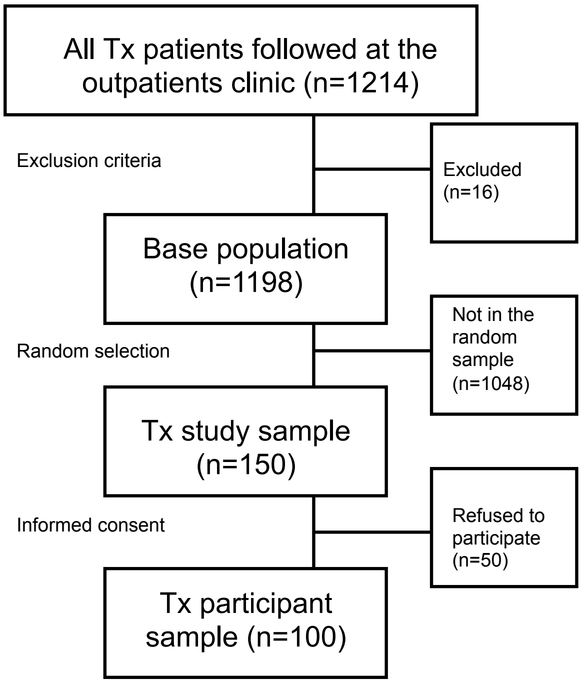

Supplement: Supplementary Information — Supplemental tables and figure [file srep06987-s1.pdf]
